# The therapeutic effect of mesenchymal stem cells on pulmonary myeloid cells following neonatal hyperoxic lung injury in mice

**DOI:** 10.1186/s12931-018-0816-x

**Published:** 2018-06-08

**Authors:** Ali Al-Rubaie, Andrea F. Wise, Foula Sozo, Robert De Matteo, Chrishan S. Samuel, Richard Harding, Sharon D. Ricardo

**Affiliations:** 1 Department of Anatomy and Developmental Biology, Biomedicine Discovery Institute, Monash University, Clayton, VIC 3800 Australia; 2 Department of Pharmacology, Biomedicine Discovery Institute, Monash University, Clayton, Australia

**Keywords:** Alveolar macrophages, Neonatal hyperoxia, Mesenchymal stem cells

## Abstract

**Background:**

Exposure to high levels of oxygen (hyperoxia) after birth leads to lung injury. Our aims were to investigate the modulation of myeloid cell sub-populations and the reduction of fibrosis in the lungs following administration of human mesenchymal stem cells (hMSC) to neonatal mice exposed to hyperoxia.

**Method:**

Newborn mice were exposed to 90% O_2_ (hyperoxia) or 21% O_2_ (normoxia) from postnatal days 0–4. A sub-group of hyperoxia mice were injected intratracheally with 2.5X10^5^ hMSCs. Using flow cytometry we assessed pulmonary immune cells at postnatal days 0, 4, 7 and 14. The following markers were chosen to identify these cells: CD45^+^ (leukocytes), Ly6C^+^Ly6G^+^ (granulocytes), CD11b^+^CD11c^+^ (macrophages); macrophage polarisation was assessed by F4/80 and CD206 expression. hMSCs expressing enhanced green fluorescent protein (eGFP) and firefly luciferase (fluc) were administered via the trachea at day 4. Lung macrophages in all groups were profiled using next generation sequencing (NGS) to assess alterations in macrophage phenotype. Pulmonary collagen deposition and morphometry were assessed at days 14 and 56 respectively.

**Results:**

At day 4, hyperoxia increased the number of pulmonary Ly6C^+^Ly6G^+^ granulocytes and F4/80^low^CD206^low^ macrophages but decreased F4/80^high^CD206^high^ macrophages. At days 7 and 14, hyperoxia increased numbers of CD45^+^ leukocytes, CD11b^+^CD11c^+^ alveolar macrophages and F4/80^low^CD206^low^ macrophages but decreased F4/80^high^CD206^high^ macrophages. hMSCs administration ameliorated these effects of hyperoxia, notably reducing numbers of CD11b^+^CD11c^+^ and F4/80^low^CD206^low^ macrophages; in contrast, F4/80^high^CD206^high^ macrophages were increased. Genes characteristic of anti-inflammatory ‘M2’ macrophages (*Arg1, Stat6, Retnla, Mrc1, Il27ra, Chil3,* and *Il12b*) were up-regulated, and pro-inflammatory ‘M1’ macrophages (*Cd86, Stat1, Socs3, Slamf1, Tnf, Fcgr1, Il12b, Il6, Il1b,* and *Il27ra)* were downregulated in isolated lung macrophages from hyperoxia-exposed mice administered hMSCs, compared to mice without hMSCs. Hydroxyproline assay at day 14 showed that the 2-fold increase in lung collagen following hyperoxia was reduced to control levels in mice administered hMSCs. By day 56 (early adulthood), hMSC administration had attenuated structural changes in hyperoxia-exposed lungs.

**Conclusions:**

Our findings suggest that hMSCs reduce neonatal lung injury caused by hyperoxia by modulation of macrophage phenotype. Not only did our cell-based therapy using hMSC induce structural repair, it limited the progression of pulmonary fibrosis.

**Electronic supplementary material:**

The online version of this article (10.1186/s12931-018-0816-x) contains supplementary material, which is available to authorized users.

## Background

Preterm infants experience a range of debilitating health issues primarily resulting from lung immaturity, and few treatment options are available [1–3]. Owing to lung underdevelopment, preterm infants often require mechanical ventilation with hyperoxic gas in order to survive [4, 5]. However, high levels of oxygen or prolonged use of ventilators can damage the lungs and interrupt normal alveolar and bronchiolar development, which may lead to chronic lung diseases known as bronchopulmonary dysplasia (BPD) [6]. It is common, that preterm infants that are born at less than 32 weeks of gestation have increased progression of other short and long-term respiratory illnesses, such as asthma and chronic obstructive pulmonary disease (COPD) [7].

Experimental studies have attempted to reduce the negative effects of hyperoxia on the developing lungs; however, clinical translation has been disappointing to date. Consequently, there is still no effective treatment for BPD in preterm infants [8–10]. BPD is associated with the inflammation of lungs, which typically involves the recruitment of monocytes that differentiate into alveolar macrophages [11]. Macrophages are a heterogeneous cell type that can be broadly categorised into two groups: “classically activated” M1 macrophages that have pro-inflammatory functions and M2 macrophages are “alternatively activated” cells that play a reparative or regulatory role [12–14].

Human mesenchymal stem cells (hMSCs) have the ability to alter macrophage phenotype from an inflammatory to anti-inflammatory phenotype that may be therapeutically beneficial to treat injured lungs [15]. There have been completed or ongoing clinical trials involving cell therapies that delay the progression or reverse a variety of immune and non-immune diseases in the lung and other organs [16]. Preclinical and clinical data support the use of cell therapy to treat COPD, acute adult lung injury and severe chronic asthma [17]. Recently, the protective effects of stem cells in lung diseases, for example BPD, have been demonstrated [18]. Studies have shown that stem cells protect the newborn injured lung from BPD and aid in endogenous repair of the injured lung tissue, either by differentiation into lung parenchymal cells [19], or into type II alveolar epithelial cells [20], via the secretion of factors [21]. However, some limitations to the studies have also been reported such as risks associated with possible immune reactions against hMSCs [22]. Understanding the therapeutic benefit of hMSCs in neonatal hyperoxic lung injury in mice remaining largely unclear, specifically, the ability to alter the lung immune cells population/s.

This study aimed to determine the effectiveness of human bone marrow-derived hMSC therapy on neonatal hyperoxia, through the modulation of pulmonary immune cells and lung injury–induced collagen deposition in mice, based on the alteration of macrophage phenotype. We showed that hMSCs tracked to the injured lung following intratracheal injection where they modulated macrophage phenotype leading to reduced collagen deposition, thus improving lung structure.

## Methods

### Experimental animals

This study was performed using pups at embryonic day 14 timed-pregnant C57BL/6 J mice obtained from the Monash Animal Research Platform (MARP), housed at Monash University, Clayton. The study was conducted under animal ethics number MARP2014/092 issued by Monash Animal Ethics Committee. Pups were born at term, and the day designated as postnatal day 0. The exposure of lungs to 90% O_2_ was used to induce injury and the effectiveness of hMSC treatment on hyperoxia-induced lung injury was assessed. Newborn mice were exposed to either normoxia 21% O_2_ (control group) or 90% O_2_ (hyperoxia) from postnatal day 0 to day 4. The oxygen concentration was maintained and monitored throughout the period of hyperoxia dams were rotated with non-hyperoxia dams (SWAP dams) every 24 h. The mice received standard chow ad libitum under 12/12 h day/night cycle. At day 4, sub-groups within the hyperoxia groups were injected directly into the trachea with hMSCs purchased from the Tulane Centre for Stem Cell Research and Regenerative Medicine (Tulane University, New Orleans, LA) [23] at passage four. 2.5X10^5^ hMSCs were suspended in 10 μl PBS and delivered via intratracheal injection using a 1 ml insulin syringe and a 29G hypodermic needle. Mouse lungs were collected at days 0, 4, 7 and 14.

### Bioluminescence imaging

For cell tracing of hMSCs in vivo, sub-groups of hyperoxia-exposed mice (*n* = 8) were administered 2.5X10^5^ hMSCs expressing enhanced green fluorescent protein (eGFP) and firefly luciferase (fluc) in 10 μl PBS at postnatal day 4 [23]. To image the hMSCs in vivo following delivery, anesthetized mice were injected intraperitoneally with 200 μl of D-luciferin (15 mg/ml in PBS; VivoGlo Luciferin, Promega, San Luis Obispo, CA, USA) and imaged on days 0, 1 and 3 following cell delivery using a IVIS 200 system (Xenogen, Alameda, CA, USA). The fluc luminescent signal was captured and analysed as photons/sec/cm^2^ (Living Image 3.2, Xenogen).

#### Lung immune cells preparation

##### Preparation of lung tissue

Freshly excised lungs were kept in cold FACS -fluorescence-activated cell sorting- buffer (Phosphate-buffered saline accompanied with 0.2% Bovine serum albumin, 0.02% Sodium azide and 5 mM EDTA). With surgical scissors, the main airways were removed and the lungs were cut into small portions before enzymatic digestion, for 45 min, in 1 mL of dissociation media consisting of HBSS- Hanks’ Balanced Salt Solution- (Sigma-Aldrich, St. Louis, USA) containing 3 mg/mL collagenase/dispase (Roche Applied Science, Penzberg, Germany) and 0.2 mg/mL DNase type 1 (Roche Applied Science). To eliminate red blood cells, the lung single cell suspensions were incubated in 1 mL of red blood cell lysis buffer (8.3 g/L NH4Cl, 10 mM Tris-HCl, and pH 7.5) for 1 min. Samples were filtered through a 100 μm nylon cell strainer (BD Bioscience, San Jose, USA) before markers labelling.

##### Flow-cytometry

Cells counts were performed using a Z2 Coulter Counter (Beckman Coulter, USA). 3X10^6^ cells from single cell suspensions were incubated at 4 °C for 20 min with the following fluorochrome-conjugated anti-mouse markers: anti-CD45 APC-Cy7 (clone 30-F11; Biolegend, San Diego, USA), anti-CD11b PE-Cy7 (clone M1/70; BD Biosciences), anti-CD11c Pacific Blue (clone N418; Biolegend), anti-Ly6C FITC (clone HK1.4; Biolegend), anti-Ly6G Alexa Fluor 647 (clone 1A8; Biolegend), anti-F4/80 APC (clone BM8; eBioscience), and anti-CD206 (mannose receptor; MR) Alexa Fluor 488 (clone C068C2; Biolegend). Fc receptor block (anti-CD16/32 antibody) was added to all markers cocktails. Intracellular CD206 labelling was performed using a CytoFix/CytoPerm kit (BD Biosciences, USA). After surface receptor labelling, cells were permeabilized and incubated with the marker for 30 min at 4 °C in the dark before being washed twice in 1× Perm/Wash buffer (BD Biosciences) and resuspend in FACS buffer. A BD FACS Canto II flow cytometer (BD Biosciences) was used to acquire data. Data was analysed using FlowLogic FCS analysis software (Inivai Technologies, Melbourne, Australia).

##### Alveolar macrophages total-RNA sequencing

At postnatal day14, 1X10^5^ CD45^+^CD11b^+^CD11c^+^ alveolar macrophages were sorted from mouse lungs (total *n* = 9; 3 from each treatment group) via an influx sorter (Flowcore, Monash University, Victoria, Australia). Total RNA was extracted from the sorted macrophages using RNeasy Kit following the manufacturer’s protocol (Qiagen, Hilden, Germany). The integrity of the samples was measured using the Agilent Bioanalyzer 2100 with B.02.08.SI648 (SR3) software and a microfluidics device, in conjunction the associated hardware and chemistry (Agilent Technologies, Waldbronn, Germany). Samples were used to construct Illumina sequencing libraries using the following Takara SMART-seq Ultra Low Input version 4 according to the manufacturer’s instructions (Clontech Takara SMART-Seq V4 Ultra Low Input RNA Kit for Sequencing User Manual v.01251). Libraries were quantitated using a Qubit DNA HS kit, which incorporates a double-stranded DNA-specific fluorescent dye (Invitrogen, Carlsbad CA., USA). Libraries were sized and checked for adapter contamination using the Agilent Bioanalyzer 2100 microfluidics device, in conjunction with Agilent DNA HS kits and chemistry (Agilent Technologies, Waldbronn, Germany). These libraries were sequenced using the following chemistry and conditions in NextSeq500 - High-Output SBS version 2 (Illumina 15,046,563 v02), with library concentration 1.8 pM and reading length 1X75b according to the manufacturer’s instructions. The differential gene expression was performed with the voom [24] limma package (v3.34.1) [25].

#### Lung fibrosis profile and structural changes

##### Hydroxyproline assay

On day 14, lung tissue from mouse pups (*n* = 8) were assessed for total collagen content, determined by hydroxyproline content as previously described [23] . Hydroxyproline values were then converted to collagen content by multiplying by a factor of 6.94 as hydroxyproline represents; 14.4% of the amino acid composition of collagen in most mammalian tissues [26]. This was determined using a standard curve of purified trans-4-hydroxy-L-proline (Sigma-Aldrich) and further expressed as a percentage of the dry tissue weight to yield collagen concentration.

##### Morphometric lung analysis

At day 56, lung tissue sections from mouse (*n* = 8 / group) were randomly selected from the left lung were embedded in paraffin for histological staining. Paraffin-embedded sections of lung tissue were stained with picrosirius red. Sections 5 μm thick were examined by light microscopy (X200 magnification) and five random sections were captured using a digital camera (SPOT Insight 4Meg Fire Wire Color Mosaic 14.2, Diagnostic Instruments, USA). To assess lung injury, mean linear intercept (MLI) was measured using software ImageJ, version 1.47 (Wayne Rasband, NIH, Bethesda, MD, USA). Large airways and vessels were avoided. The program placed horizontal lines, 30 μm apart, across each section of lung. Then the number of times the lines intercepted the alveoli (intercepts) was calculated. All counting methods have been described previously [27].

### Statistical analysis

Data were analysed using GraphPad Prism software version 7.0c (GraphPad Software Inc., San Diego, USA) and IBM SPSS statistics (The Apache Software Foundation). A *t*-test (unpaired, two-tailed) was used to analyse differences between the normoxia and hyperoxia groups at day 4. A one-way analysis of variance with a Tukey’s multiple comparisons test was used to analyse data contained in the normoxia, hyperoxia and hyperoxia + hMSC groups at day 7 and day 14. Data are given as means ± SEM and *P* < 0.05 was considered statistically significant.

## Results

### Bioluminescence imaging of hMSCs

**Fig. 1:**
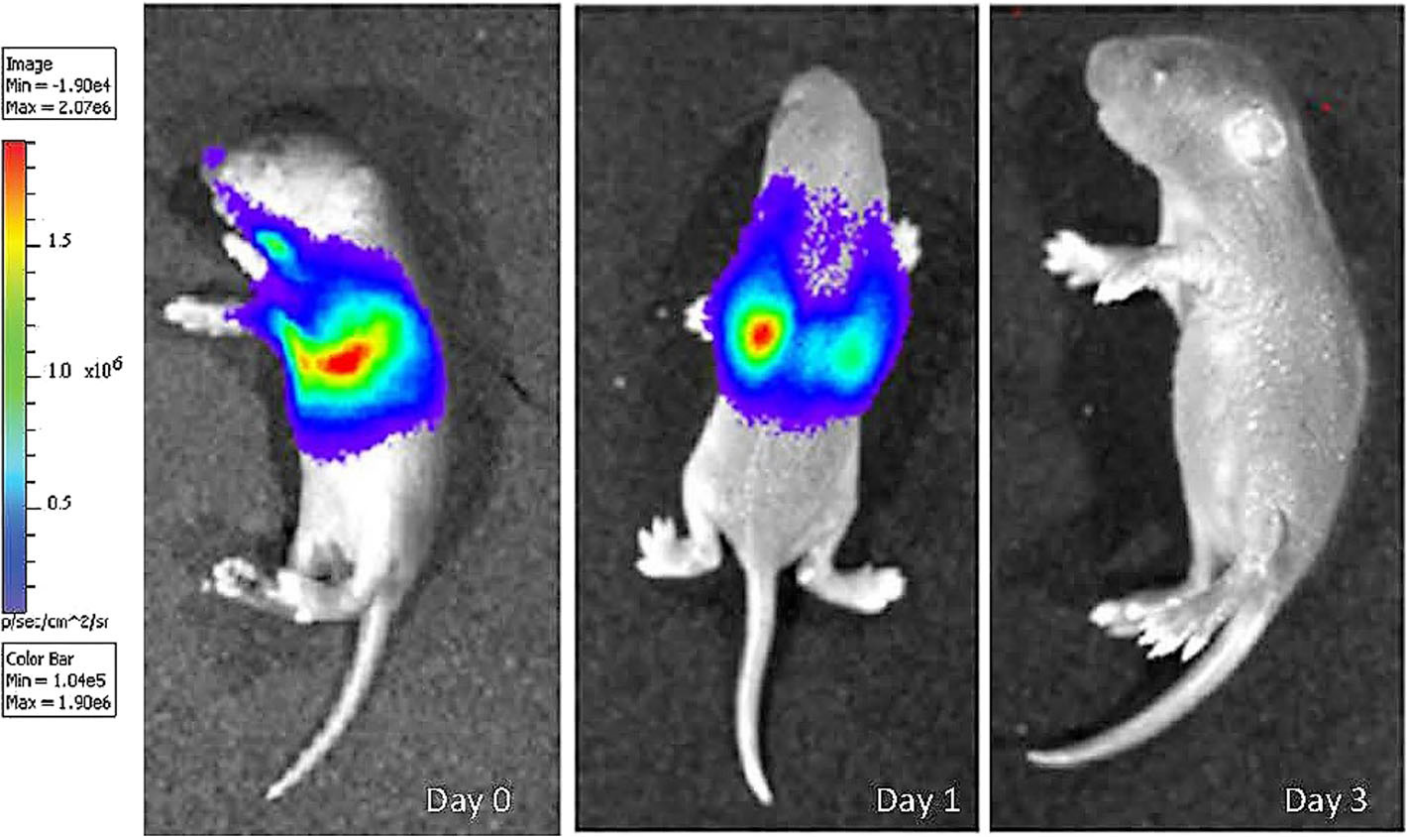
Luciferin/GFP + MSCs were injected into mice after hyperoxia *n* = 8, and the bioluminescence fluc signal was examined at time 0 and day 1 and 3 after luciferase injection. Bioluminescence imaging showing the intensity of fluc signal representing MSC localization to lungs. Red signals indicate high intensity of MSCs and blue indicates low intensity – see heatmap panel

Following intratracheal injection of hMSCs at day 4, whole body bioluminescence imaging was used to confirm the fate of the hMSCs in vivo. The intensity of bioluminescence has previously been shown to be directly proportional to the number of labelled hMSCs residing in the host tissue [28]. hMSCs administered to hyperoxia and normoxia exposed lungs were found to localize in the lungs when assessed at days 0 and 1 post-administration (Fig. 1). hMSCs were clearly detected in the thoracic area of the pups, corresponding to the position of the lungs. Bioluminescence detection of hMSCs reduced over time, whereby there was no signal by day 3.

### Flow cytometry

**Fig. 2:**
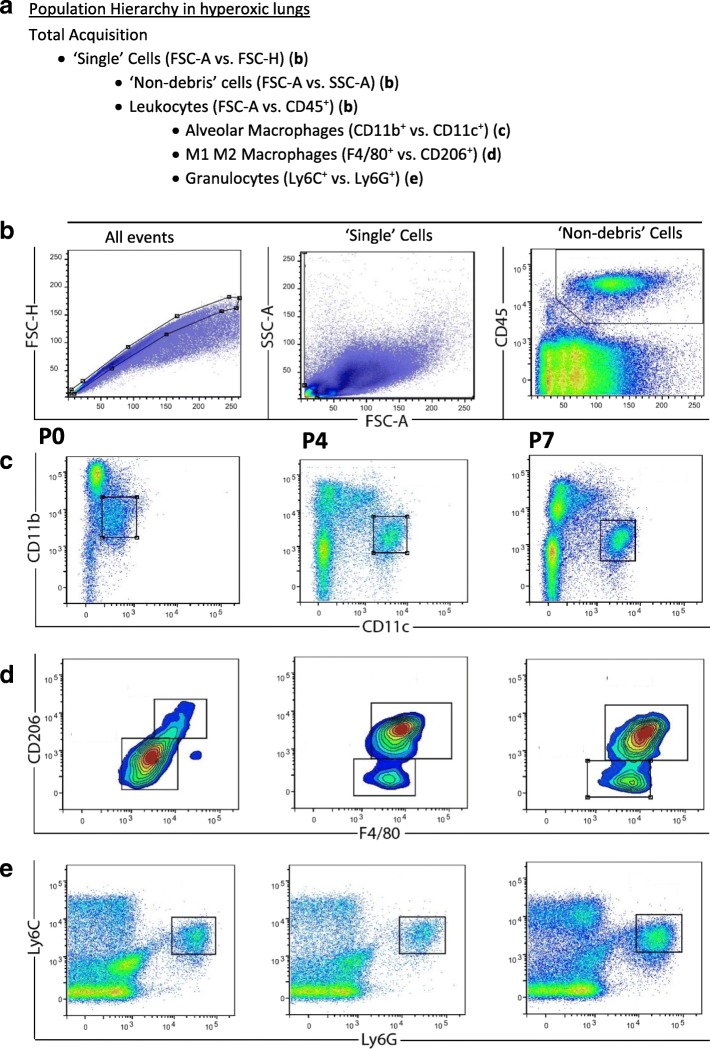
The representative gating strategy for assessing myeloid cell sub-populations in the lung. The population hierarchy shows the gating strategy (**a**). ‘Single’ cells (excluding doublets and triplets) were selected with a polygon gate on a FSC-A vs. FSC-H dot plot (**b**). Single cells were gated on the resulting daughter population on a FSC-A vs. SCA-A dot plot (**b**). A ‘Live’ cell gate (which excludes debris) was created with the aid of the CD45^+^ cells on FSC-A vs. CD45^+^ (**b**). CD45^+^CD11b^+^CD11c^+^ alveolar macrophages were selected with a gate (**b**), CD45^+^ F4/80^+^ CD206^+^ M1 vs. M2 macrophages were selected with a gate (**d**) and CD45^+^Ly6C^+^Ly6G^+^ granulocytes (**e**) were selected for further analysis of myeloid cell subsets. Plots in c, d and e are from a hyperoxic lung taken at day 0, day 4, and day 7 from left to right

Flow cytometry was performed on days 0, 4, 7 and 14 to evaluate the effect of hyperoxia and hMSC treatment on the numbers of lung CD45^+^ leukocytes and sub-populations of myeloid cells. Fig. 2 shows the gating strategy used for analysing immune cells in the lung. Immunofluorescent staining of the mononuclear cells using markers cocktail was used to identify the CD45^+^ leukocytes.

There were significant changes in the CD45^+^ leukocytes populations and the sub-populations including granulocytes populations expressing Ly6C^+^Ly6G^+^, alveolar macrophages populations expressing CD11b^+^CD11c^+^ and their sub-populations macrophages phenotypes F4/80^low^CD206^low^ ‘M1’ and F4/80^high^CD206^high^ ‘M1’among the three groups analysed.

### CD45^+^ leukocytes

**Fig. 3:**
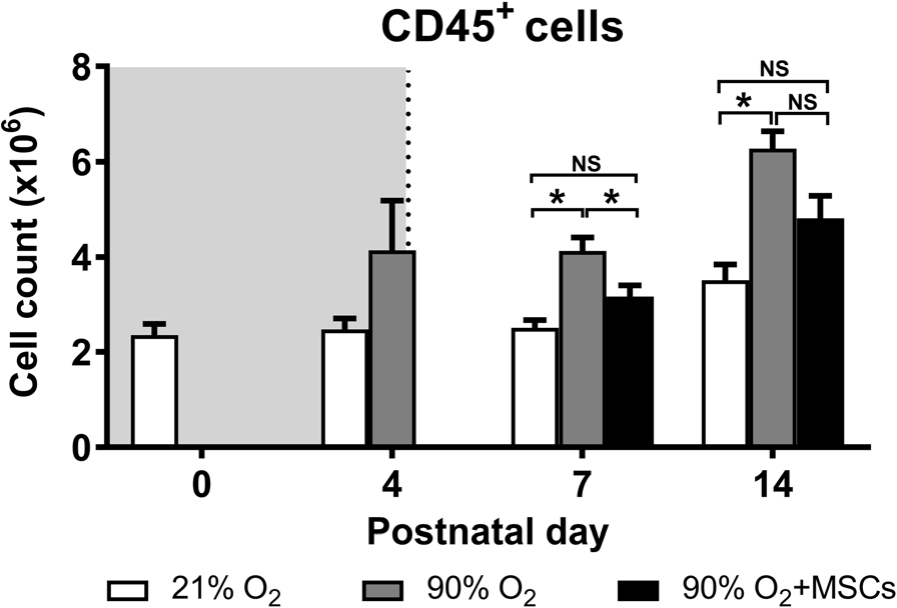
CD45^+^ myeloid cell count in lungs from mice following normoxia (), 90% O_2_ () and 90% O_2_ with administration of hMSC () delivered at day 4. The shaded area (grey) depicts period of hyperoxia exposure (postnatal days 0–4) prior to delivery of hMSCs (dotted line) on day 4. For all treatment groups, *n* = 8 per group. Data are shown as mean ± SEM. * = *p* < 0.05

At day 7 and day 14 there was a greater number of CD45^+^ cells (*P* < 0.05) in hyperoxia-exposed lungs compared to normoxia-exposed lungs (Fig. 3). Importantly, at day 7 hMSC administration to mice exposed to hyperoxia significantly reduced the number of CD45^+^ cells in the lungs compared to hyperoxia-exposed lungs (*P* < 0.05).

#### Granulocytes

**Fig. 4:**
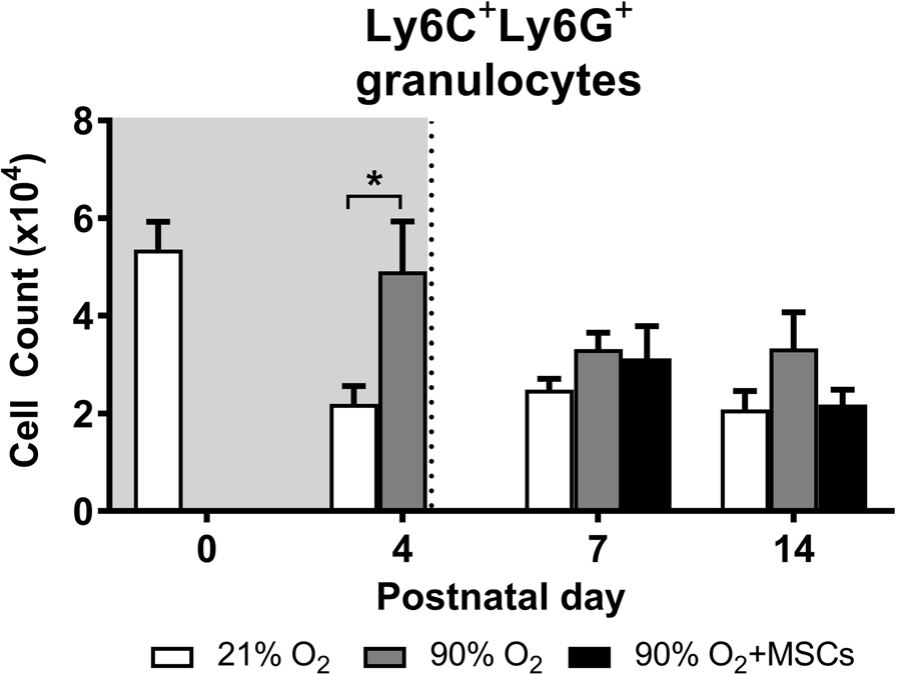
Ly6C^+^Ly6G^+^ granulocyte cell count in lungs from mice following normoxia , 90% O_2_ () and 90% O_2_ with administration of hMSC () delivered at day 4. The shaded area (gray) depicts period of hyperoxia exposure (postnatal days 0–4) prior to delivery of hMSCs (dotted line) on day 4. For all treatment groups, *n* = 8 per group. Data are shown as mean ± SEM. * = *p* < 0.05

At day 4, the number of Ly6C^+^Ly6G^+^ granulocytes were significantly higher (*P* < 0.05) in lungs from hyperoxia-exposed mice, compared to mice receiving normoxia. Interestingly, the administration of MSCs to mice with lungs exposed to hyperoxia had no impact on the numbers of granulocytes at day 7 and 14 (Fig. 4).

#### Alveolar macrophages

**Fig. 5:**
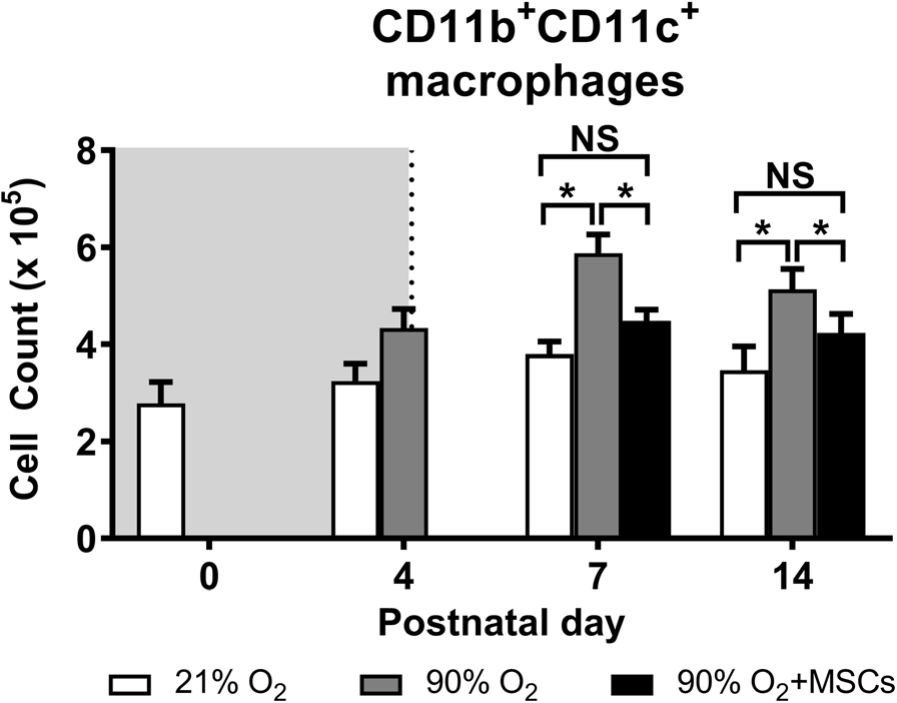
CD11b^+^CD11c^+^ macrophage cell count in lungs from mice following normoxia (), 90% O_2_ () and 90% O_2_ with administration of hMSC () delivered at day 4. The shaded area (gray) depicts period of hyperoxia exposure (postnatal days 0–4) prior to delivery of hMSCs (dotted line) on day 4. For all treatment groups, *n* = 8 per group. Data are shown as mean ± SEM. * = *p* < 0.05 and NS = not significant

**Fig. 6:**
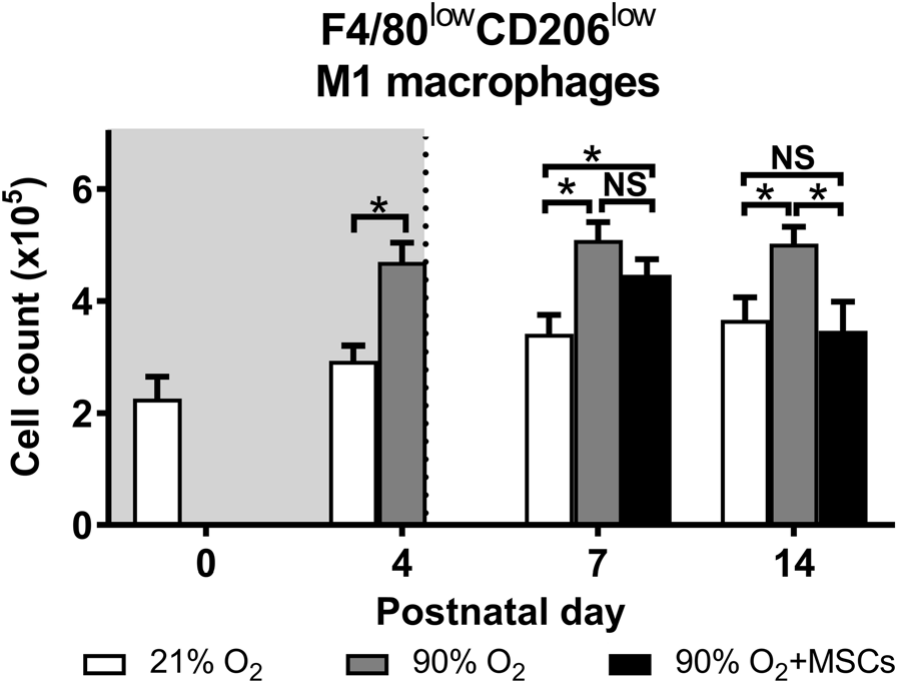
F4/80^low^CD206^low^ M1 macrophage cell count in lungs from mice following normoxia (), 90% O_2_ () and 90% O_2_ with administration of hMSC () delivered at day 4. The shaded area (gray) depicts period of hyperoxia exposure (postnatal days 0–4) prior to delivery of hMSCs (dotted line) on day 4. For all treatment groups, *n* = 8 per group. Data are shown as mean ± SEM. * = *p* < 0.05 and NS = not significant

**Fig. 7:**
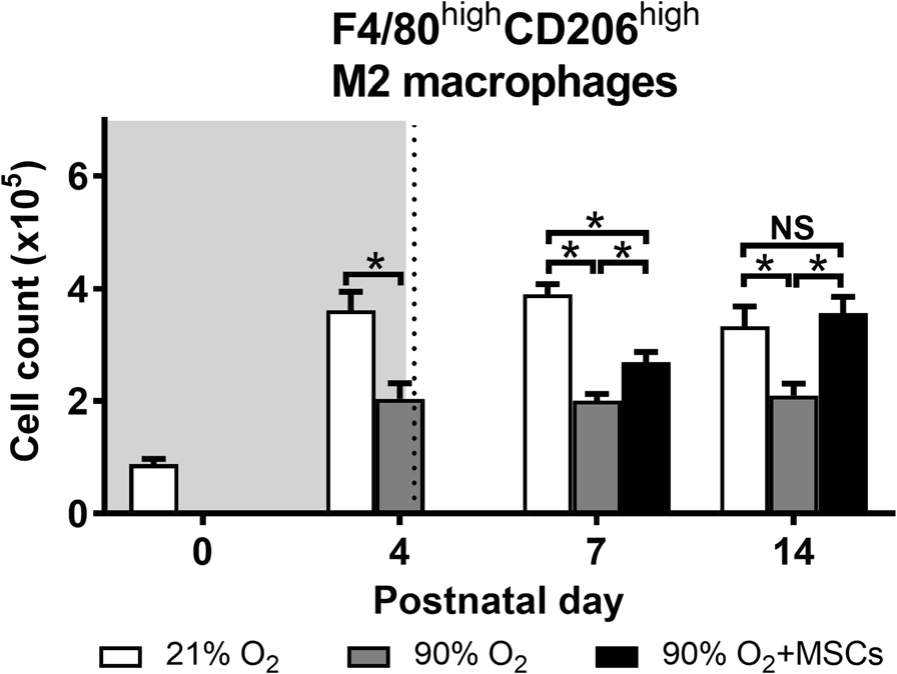
F4/80^high^CD206^high^ M2 macrophage cell count in lungs from mice following normoxia (), 90% O_2_ () and 90% O_2_ with administration of hMSC () delivered at day 4. The shaded area (gray) depicts period of hyperoxia exposure (postnatal days 0–4) prior to delivery of hMSCs (dotted line) on day 4. For all treatment groups, *n* = 8 per group. Data are shown as mean ± SEM. * = *p* < 0.05 and NS = not significant

The number of CD11b^+^CD11c^+^ alveolar macrophages, representing a sub-population of the CD45^+^ cells. At day 4, there are no significant differences between the treatment groups. However, by day 7 and 14 there were significantly more CD11b^+^CD11c^+^ macrophages in the lungs from mice exposed to hyperoxia compared to normoxia (*P* < 0.05). In comparison, hMSC administration starting at day 4 was effective in reducing the number of alveolar macrophages at day 7 and 14, comparable to the normoxia group (*P* < 0.05; Fig. 5). From the CD11b^+^CD11c^+^ alveolar macrophages, two sub-populations –phenotypes– were examined using F4/80 and CD206 expression:

- ***M1 macrophages***
  The number of F4/80^low^CD206^low^ macrophages sub-population, that represent an inflammatory ‘M1’ phenotype, significantly changed among the treatment groups. Hyperoxia caused a significant rise in the number of F4/80^low^CD206^low^ macrophages at days 4, 7 and 14, compared to the normoxia group (*P* < 0.05; Fig. 6). This increase in cell number at day 14 was attenuated following the hMSC treatment, where there was no significant difference between normoxia and hMSC injected hyperoxia groups.
- ***M2 macrophages***
  The number of F4/80^high^CD206^high^ cells, that represent an anti-inflammatory ‘M2’ phenotype, decreased in mice exposed to hyperoxia compared to normoxia over all time points studied (*P* < 0.05; Fig. 7). At day 4, hyperoxia exposure resulted in a significant decrease in the number of F4/80^high^CD206^high^ cells in mouse lungs compared to the normoxia group (*P* < 0.05). At day 7 and 14, hMSC administration was able to effectively increase (*P* < 0.05) the number of F4/80^high^CD206^high^ M2 macrophages. At day 14, the cell number was restored to control levels where there was no significant difference between normoxia and hMSC injected hyperoxia groups.

### Alveolar macrophages total- RNA sequencing

Using next generation sequencing, macrophage total-RNA gene expression was analysed assessing specific genes characteristic of M1- proinflammatory macrophages including *Cd86, Stat1, Socs3, Slamf1, Tnf, Fcgr1, Il12b, Il6, Il1b,* and *Il27ra*. These genes were up-regulated in isolated lung macrophages from mice exposed to hyperoxia compared to mice receiving hMSCs. Furthermore, genes related to M2- anti-inflammatory macrophages showed overall down regulation in macrophages isolated from lungs of hyperoxic mice receiving hMSCs. These genes included *Arg1, Stat6, Retnla, Mrc1, Il27ra, Chil3,* and *Il12b* [29], as shown in Additional file 1: Table S1.

### Collagen accumulation is decreased by hMSCs in hyperoxic mouse lungs

**Fig. 8:**
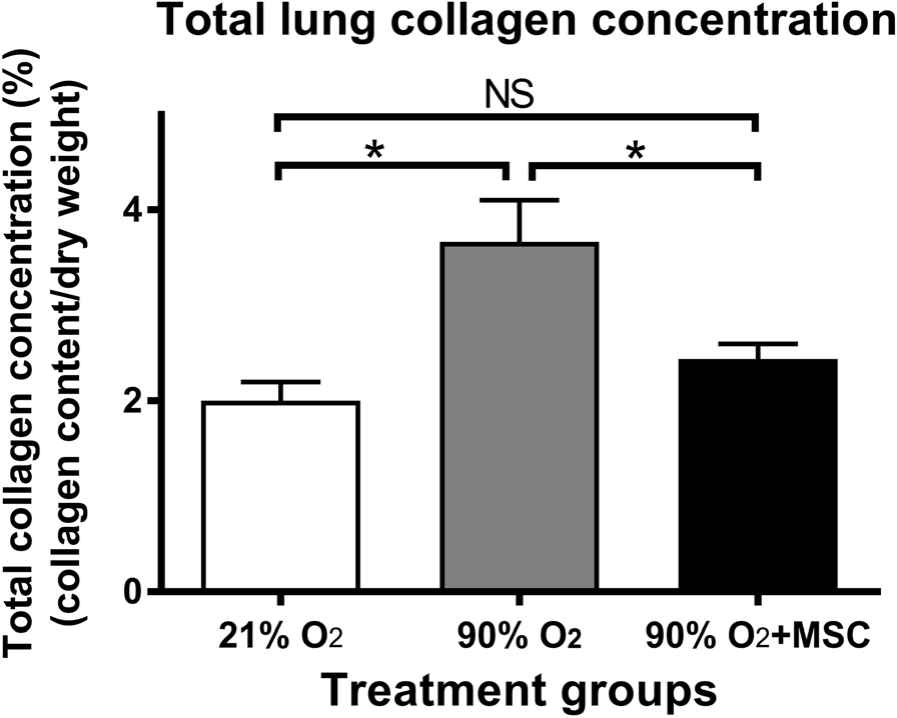
hMSCs reduce collagen accumulation in the lung following hyperoxic injury total lung collagen concentration (% collagen content/dry weight tissue) in normoxia, 90% O_2_ and 90% O_2_ with administration of hMSC on postnatal day 14. For all treatment groups *n* = 8. Data are shown as mean ± SEM. * = *p* < 0.05 and NS = not significant

Hydroxyproline analysis of total collagen concentration was used to assess the progression of lung fibrosis in mouse lungs exposed to either normoxia or hyperoxia in comparison to mice exposed to hyperoxia under the treatment of hMSCs (Fig. 8). Mice exposed to hyperoxia showed a 2 fold increase in lung collagen accumulation (percent collagen content per dry weight tissue) at day 14 compared with the normoxia animals. There was also a significant reduction in total collagen content, representing reduced interstitial fibrosis, in lungs from hMSC-treated mice exposed to hyperoxia. The collagen content in these mice was comparable to control mice.

### Lung structure improves in hyperoxic mice following hMSC delivery

**Fig. 9:**
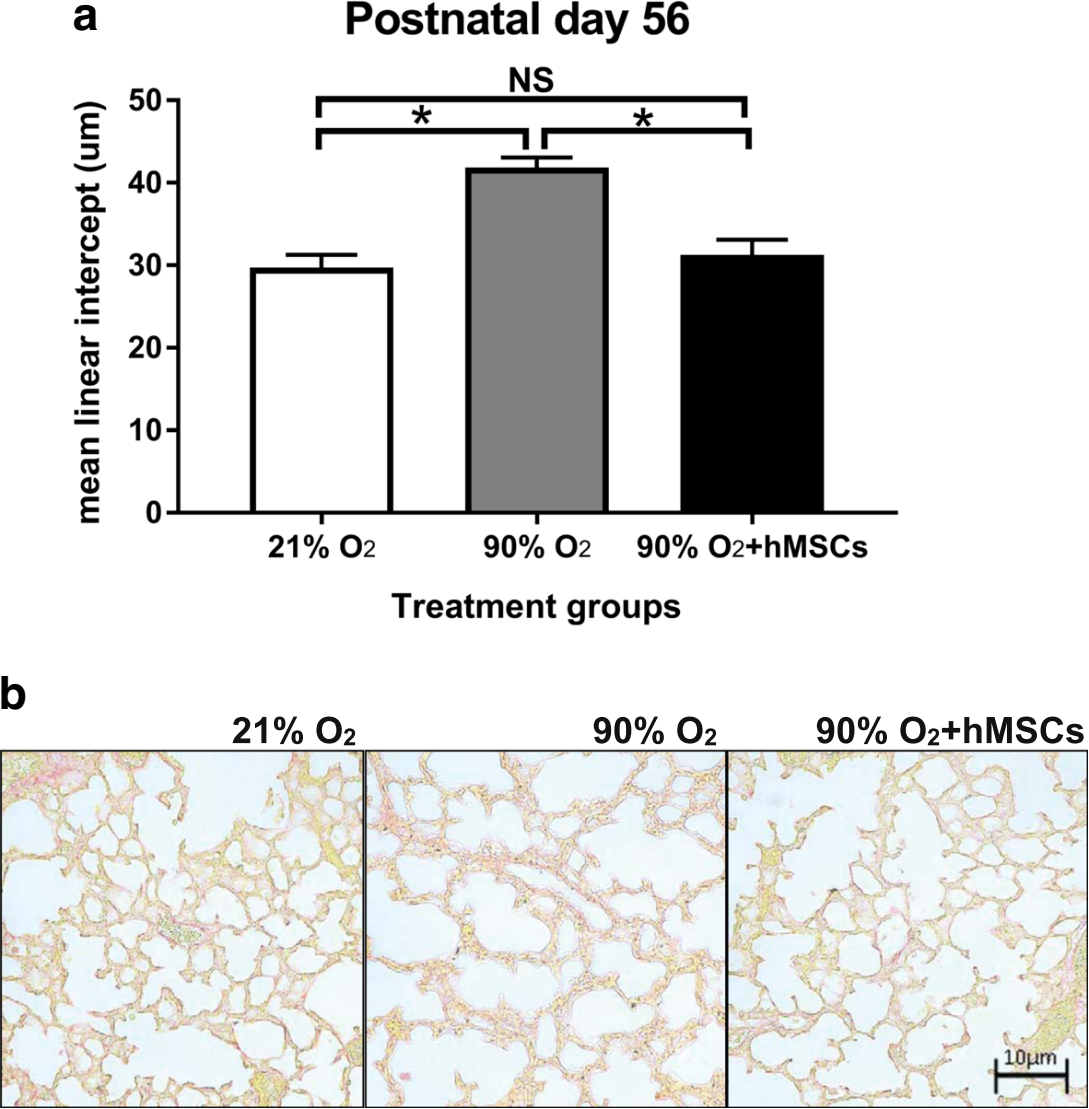
**a** MLI following normoxia, 90% O_2_ () and 90% O_2_ + hMSC (). 90% O_2_ was given between postnatal days 0–4 and MSCs were given on postnatal day 4. For all treatment groups at day 56 (*n* = *8* per group). Data were analysed using a one-way ANOVA and shown as mean ± SEM. *p* < 0.05. **b** representative histological images of the three treatment groups stained with picrosirius red

The assessment of lung histology showed that the administration of hMSC to mice exposed to hyperoxia improved lung architecture and alveolar structure (Fig. 9b), which was consistent with an increased MLI (Fig. 9a). At day 56, MLI was higher in hyperoxia treated mouse lungs 41.84 ± 0.39 μm) compared to the lungs of mice exposed to normoxia (29.74 ± 0.48 μm). However, the hyperoxia+hMSC group showed a noticeable amelioration of injury as evidenced by a decrease in the MLI compared to hyperoxia without administration of hMSC (31.27 ± 0.56 μm vs. 41.84 ± 0.39 μm; *P* < 0.05). The MLI from lungs of normoxic mice was not significantly different from the MLI of mice exposed to hyperoxia administered MSCs.

## Discussion

The administration of hMSCs to the neonatal lung ameliorated the hyperoxia-induced injuries, including reducing collagen deposition. Additionally, the hMSC administration was found to effectively reduce the hyperoxia-induced infiltration and phenotype of sub-populations of macrophages into the damaged lung.

To study the effects of hMSC therapy in the neonatal lung, a mouse model was used which mimics the effects of neonatal lung hyperoxia in human preterm babies [1]. This model has provided significant insights into the lung pathology induced by exposing the developing lungs to hyperoxic gas [30]. This study provides the first evidence that a non-surgical, intra-airway route of administration in mice can effectively deliver hMSCs to the neonatal lung as early as one-hour post-injection where they remained elevated for 24 h. Confirmation of MSCs in damaged lungs has been difficult to ascertain due to the entrapment of hMSCs in lung capillaries when delivered intravenously [31].

The exposure of neonatal mice to 90% O_2_ induced lung injury by postnatal day 14, where there was an accumulation of interstitial collagen which is consistent with a previous report [32]. This finding was associated with pathological changes to the lungs, namely alveolar wall thickening and pathological changes indicative of emphysema [30].

The current study used polychromatic flow cytometry analysis to identify and compare granulocytes and macrophage phenotypes in the normoxic lung to the inflammatory lung following hyperoxic injury.

Our hypothesis on the lung response to hyperoxic injury was assessed by quantifying the inflammatory cells, a method we could study by using the flow cytometric assay.

In this study, CD45^+^ leukocytes were used to quantify the two subsets of granulocytes and macrophages without reference to the other leukocyte subsets (natural killer cells, invariant natural killer T-cell, T-helper cell, Cytotoxic T-cell, Dendritic cell, monocytes) [33].

The use of separate markers against Ly6C and Ly6G allowed a defined demarcation of granulocytes from other CD45 myeloid cells populations [34]. Furthermore, we used the standard approach of staining with both CD11b and CD11c markers to differentiate macrophages from other myeloid cell populations [33, 34]. The increased proportion of granulocytes indicated an inflammatory response following four days of exposure to hyperoxia, which was consistent with studies showing that granulocytes are the predominant cell type that infiltrates the lung tissue following an injury [35, 36]. The inflammatory environment may provide important cues leading to the infiltration of other inflammatory cells, including blood monocytes, that have the propensity to differentiate into M1 and M2 macrophages [37].

We have shown that hMSC attenuates the increase in the total number of CD45^+^ leukocyte (*P* < 0.05) at day 7 in the neonatal lung following 4 days of exposure to hyperoxia. The elevation of the leukocytes occurred as a result of primary granulocytes recruitment into the alveolar spaces and pulmonary interstitial parenchyma, as defined previously in different lung injury literature [38].

In the present study, the cell count of Ly6C^+^Ly6G^+^ granulocytes in lung tissue was elevated at day 4 in the mice exposed to hyperoxia and reduced thereafter. Moreover, the administration of hMSCs was shown to have no effect of granulocyte number in hyperoxic lungs at day 7 and day 14. However, other studies suggest that the granulocytes might raise as a result of releasing granulocyte colony stimulating factor from the stem cells [39].

This study showed that the CD11b CD11c expressed macrophages were relatively high from day zero, although the level of CD11c expression was low in comparison with day 4 and 7 as shown in a representative FACS plots in Fig. 2. It is hard to confirm whether these macrophages are monocytes derived macrophages or foetal monocytes-macrophages, and immature-macrophages, with significant overlap in expression of marker sets. These interstitial macrophages were derived from the yolk sac [12] which initially derived from mesoderm cells that develop during gastrulation from the primitive streak [40].

Throughout the study, we demonstrated that CD11b^+^CD11c^+^ macrophages increased in response to hyperoxia by day 14, including an increased population of F4/80^low^CD206^low^ inflammatory ‘M1’ macrophages and decreased F4/80^high^CD206^high^ anti-inflammatory ‘M2’ macrophages. In other studies, M1 macrophage infiltration has been confirmed as well, together with myeloid differentiation and the alteration of relative function at the site of inflammation [41, 42]. These changes in phenotype result in the activation of monocytes at different maturation stages leading to mature macrophages of distinctive functional states [43]. Because macrophages are necessary for the phagocytosis of apoptotic neutrophils, the exposure to neonatal hyperoxia may lead to a reduced number of macrophages proceeding to necrosis, leading to the expansion of damaged alveoli [11]. This conclusion contradicts studies suggesting that macrophages might secrete certain chemokines, which could influence neutrophil infiltration and recruitment [11].

Our data confirms the beneficial effect of hMSC treatment on the phenotype and function of macrophages after neonatal hyperoxia. The administration of MSCs to mice with hyperoxia-induced acute lung injury was shown alteration of the macrophages proportion and phenotypes. These findings indicated the presence of an inflammatory environment. These results have also been demonstrated in an experimental model of asthma after bone marrow-derived hMSC administration [44].

Following the intra-airway administration of hMSCs, an increase in M2 macrophages was observed in the injured lungs of mice. It is possible that the reduction in lung injury in hMSC-treated mice was due to downregulation of proinflammatory factors and the upregulation of anti-inflammatory chemokines. In contrast, the advantageous effects of hMSC treatment were displayed by reducing eosinophil infiltration in mouse models with allergic lung injury [45]. These findings and other supporting studies suggested that hMSCs are able to alter the balance of macrophage phenotype and function that occur during injury to promote repair [46]. We showed that the protective effect of hMSCs in hyperoxia-induced lung injury could alter macrophage phenotype. Recent studies have shown the in vivo interaction between hMSCs and macrophages to promote M2 polarisation. The **in vitro** co-culture of hMSCs and macrophages resulted in an alternatively activated macrophage phenotype characterised as mannose receptor (MR) high, IL-10high, IL-6high, TNF-αlow and IL-12low which exhibits enhanced phagocytic activity, increased secretion of IL-10 and VEGF and decreased secretion of pro-inflammatory cytokines [44]. Further investigation to identify the polarisation mechanism will be important for the understanding how hMSCs alter the host response following therapeutic delivery, in the context not only the inflammatory response to hyperoxia, but also lung disorders such as allergic asthma and COPD. Apart from the alteration of lung myeloid cells, lung fibrosis is the final common pathway of lung injury regardless of aetiology. In examining the effect of hMSCs therapy on lung injury induced by 90% O_2_, we chose an end-point of delivery of oxygen of four days as published data has shown that delivery of hMSCs may ameliorate established lung fibrosis after 14 days of hyperoxia- induced injury [47]. We found that the treatment with hMSCs induced decreased lung collagen accumulation following hyperoxia. We propose that hMSCs are able to create a more favourable environment, leading to less tissue damage and fibrosis. In this environment, hMSCs may possibly not only alter lung immune cells but may also have an increased capacity to facilitate their antifibrotic and reparative effects, resulting in a greater reduction in fibrosis [48]. The administration of hMSCs to the neonatal lung ameliorated the hyperoxia-induced structural injuries, including reducing collagen deposition. Additionally, the intratracheal delivery of hMSC was found to effectively reduce the hyperoxia-induced infiltration of myeloid cells, including sub-populations of macrophages, into the damaged lung tissue. This indicates that hMSCs can modulate the inflammatory environment in the lung to reduce the development of fibrotic damage and structural injury.

Using hMSCs in a wide range of settings has shown impressive treatment responses as these cells release anti-inflammatory factors providing a positive effect by modulating the inflammatory environments, hence improving tissue healing [46]. Our study has shown the effect of the administration of hMSCs immediately, after exposure to hyperoxia, on myeloid cell sub-populations in the lung. Given our findings, it is possible that future clinical trials may incorporate the use of hMSCs therapy to reduce the lung injury including injury that may developed following in preterm birth hyperoxia and other lung diseases that results from pathologic fibrosis. This study was designed to test the efficacy of hMSCs in limiting hyperoxia-induced lung injury. In order to establish a ‘proof of principle’ we used 90% oxygen, which is a higher concentration of oxygen that is used clinically. It is recognised that our hyperoxic lung injury mouse model is likely to induce a more severe form of BPD than in preterm infants in normal physiological condition. In the future, it will be critical to assess the effectiveness of hMSC in lung injury models that more closely replicate clinical conditions. Additionally, the exposure to 90% O_2_ is likely to cause hyperoxic injury in organs other than the lungs. As hMSCs administered intratracheally will likely home to injured tissues, it will be important study the systemic effects of hMSC in future studies.

## Conclusions

Our study provides evidence for a novel cellular-based therapy that can induce lung repair and limit the progression of fibrosis in neonatal lungs exposed to hyperoxic gas. The results from this study demonstrate that hMSC therapy may provide improved therapeutic options for preterm infants necessarily exposed to hyperoxic gas, and may therefore reduce the risk of COPD and asthma in later life.

## Additional file

Additional file 1: **Table S1.** Table showing the most differentially expressed genes in isolated macrophages from lungs of mice (*n* = 9) exposed to normoxia or hyperoxia with/without administration of hMSCs. RNA was isolated, then analysed, using next generation sequencing. The genes that are related to M1 and M2 macrophages were either up-regulated or down-regulated in isolated lung macrophages in response to hyperoxia, that were altered following delivery of hMSCs. Abbreviations: Cd86 (Cluster of Differentiation 86), Socs3 (suppressor of and cytokine signalling), Slamf1 (signalling lymphocytic activation molecule family member), Tnf (tumour necrosis factor), Fcgr1 (Fc receptor, IgG, low affinity), Il12b (Interleukin 12B), Il1b (Interleukin 1 beta), Il6 (Interleukin 6), Arg1 (arginase enzyme gene), Stat6 (Signal transducer and activator of transcription 6) Retnla (resistin like alpha), Mrc1 (Mannose Receptor C-Type 1), Il27ra (Interleukin 27 Receptor Subunit Alpha), Il12b (Interleukin 12B). Data showing the Log2-Fold change in gene expression between the treatments groups (false discovery rate FDR *P*-value < 0.05) for each expression. (PDF 16 kb)

## Abbreviations

BPD: Bronchopulmonary dysplasia
COPD: Chronic Obstructive Pulmonary Disease
eGFP: Enhanced Green Fluorescent Protein
FACS: Fluorescence-activated cell sorting
FMO: Fluorescence minus one
HBSS: Hanks’ Balanced Salt Solution.
hMSCs: Human mesenchymal stem cells
MLI: Mean linear intercept
NGS: Next generation sequencing
SEM: Standard error of the mean
