# Supplementary material for: The therapeutic effect of mesenchymal stem cells on pulmonary myeloid cells following neonatal hyperoxic lung injury in mice
Source: Respir Res. 2018 Jun 8;19:114. doi: 10.1186/s12931-018-0816-x (PMC5994120; doi:10.1186/s12931-018-0816-x)
Supplement: Supplementary file 1 — Table S1. Table showing the most differentially expressed genes in isolated macrophages from lungs of mice (n = 9) exposed to normoxia or hyperoxia with/without administration of hMSCs. RNA was isolated, then analysed, using next generation sequencing. The genes that are related to M1 and M2 macrophages were either up-regulated or down-regulated in isolated lung macrophages in response to hyperoxia, that were altered following delivery of hMSCs. Abbreviations: Cd86 (Cluster of Differentiation 86), Socs3 (suppressor of and cytokine signalling), Slamf1 (signalling lymphocytic activation molecule family member), Tnf (tumour necrosis factor), Fcgr1 (Fc receptor, IgG, low affinity), Il12b (Interleukin 12B), Il1b (Interleukin 1 beta), Il6 (Interleukin 6), Arg1 (arginase enzyme gene), Stat6 (Signal transducer and activator of transcription 6) Retnla (resistin like alpha), Mrc1 (Mannose Receptor C-Type 1), Il27ra (Interleukin 27 Receptor Subunit Alpha), Il12b (Interleukin 12B). Data showing the Log2-Fold change in gene expression between the treatments groups (false discovery rate FDR P-value < 0.05) for each expression. (PDF 16 kb) [file 12931_2018_816_MOESM1_ESM.pdf]

Lung Macrophages Genes	Log_2 Fold change			
	Normoxia	Hyperoxia	Hyperoxia -hMSCs	*P* value
**Cd86 (M1)**	0	+1.23	+0.24	5.12
**Socs3 (M1)**	0	+0.81	+0.42	2.02
**Slamf1 (M1)**	0	+0.22	+2.05	0.20
**Tnf (M1)**	0	+0.33	-0.42	2.31
**Fcgr1 (M1)**	0	-0.18	-0.31	0.08
**Il12b (M1)**	0	+0.56	-0.10	5.52
**Il1b (M1)**	0	+0.29	-0.19	5.60
**Il6 (M2)**	0	-0.76	-1.32	4.46
**Arg1 (M2)**	0	-0.26	+0.83	0.16
**Stat6 (M2)**	0	+0.75	+0.84	1.06
**Retnla (M2)**	0	-1.91	-1.52	6.73
**Mrc1 (M2)**	0	+0.58	+0.44	9.33
**Il27ra (M2)**	0	+0.28	-0.21	0.15
**Il12b (M2)**	0	+0.56	-0.10	5.52
